# Dynamic Equivalent Resistance Model of Knitted Strain Sensor under In-Plane and Three-Dimensional Surfaces Elongation

**DOI:** 10.3390/polym14142839

**Published:** 2022-07-12

**Authors:** Yutian Li, Pibo Ma, Mingwei Tian, Miao Yu

**Affiliations:** 1 College of Textiles and Clothing, Qingdao University, Qingdao 266071, China; liyutian@qdu.edu.cn; 2 Engineering Research Center for Knitting Technology, Ministry of Education, Jiangnan University, Wuxi 214122, China; mapibo@jiangnan.edu.cn; 3 China National Textile and Apparel Council Key Laboratory of Flexible Devices for Intelligent Textile and Apparel, Soochow University, Suzhou 215123, China

**Keywords:** knitted sensor, equivalent resistance, topology model, volume resistance, three-dimensional

## Abstract

The dynamic equivalent resistance is a major index that determines the sensing performance of knitted strain sensors, and has the characteristics of in-plane and three-dimensional curved strain sensing. Therefore, in addition to establishing the in-plane equivalent resistance, it is necessary to establish a three-dimensional equivalent resistance model to fully explain the surface sensing performance. This project establishes two equivalent resistance models of knitted strain sensors under in-plane deformation and one equivalent resistance model of three-dimensional curved surface strain. Based on the length of resistance and the geometric topological structure, an in-plane strain macro–micro equivalent resistance model and a topological equivalent resistance model are established, respectively. In addition, a three-dimensional curved surface equivalent resistance model is created based on the volume resistance. By comparing the theoretical model with the experimental data, the results prove that the proposed in-plane and three-dimensional models can be utilized to calculate the resistance change of knitted strain sensors. Length resistance, coil transfer, and curved surface deformation depth are the main factors that affect the equivalent resistance of knitted strain sensors.

## 1. Introduction

The application of wearable strain sensors in detecting body motion posture, exercise rehabilitation, and healthcare makes our life much convenient [1,2,3,4]. Knitting fabrics, due to their multifunctional characteristics in terms of elasticity, flexibility, and deformability, are suitable to be employed in wearable garments as body monitoring sensors for movement [5,6,7]. Compared to woven and non-woven fabrics, knitting fabrics have unique properties [8,9,10,11,12]. Most textile-based sensors are based on the change in resistance [13,14,15,16], which can be used to predict fabric deformation and mechanical properties [17,18,19]. Therefore, there is a huge need to study the basic electro-mechanical properties of conductive knitted fabric, and develop the application of knitted strain sensors [20,21,22,23,24].

In the study of conductive fabric, some authors have studied the equivalent resistance model of textile sensors in their original state and under uniaxial stretching [22,25,26]; for example, Zhao et al. calculated and predicted the resistance of woven conductive fabric through the radius of the warp yarn and the resistance of one unit of conductive yarn [27]. Li et al. [28,29] explored the electro-mechanical properties of conductive yarn and conductive knitted fabric, with results given in terms of length-related resistance. It was shown that the resistance related to the length plays a dominant role in the total equivalent resistance [30]. However, the resistance change of the loop transfer was not mentioned [31]. Wang et al. examined the electro-mechanical property of conductive elastic knitted fabric based on a loops structure under biaxial extensions. Tokarska evaluated the planar anisotropy of conductive knitted fabrics and determined the resistance value on the surface of the knitted fabrics [32,33]. However, the fact is that in the process of stress deformation of conductive knitted fabric, which is affected by many factors such as environmental friction, both macroscopic fabric deformation and microscopic loop resistance changes should be considered.

The strain knitted sensor must consider the superimposed resistance problem of mutual contact between the coils in the initial state [34,35]. Therefore, the superimposed resistance of the loops squeezing against each other also plays an important role when the resistance changes [36,37,38,39]. Few studies can take into account the close contact resistance of the knitted loops in the initial state, and no appropriate theoretical model is given for exploring the equivalent resistance model of the knitted sensor under the initial state strain [40]. To explore the in-plane tensile properties, several papers have described corresponding equivalent resistance models under uniaxial and biaxial elongation and verified the validity of the corresponding model [41,42,43,44,45]. However, there are limited studies on the equivalent resistance model based on three-dimensional curved surface strain sensing.

**Figure 1:**
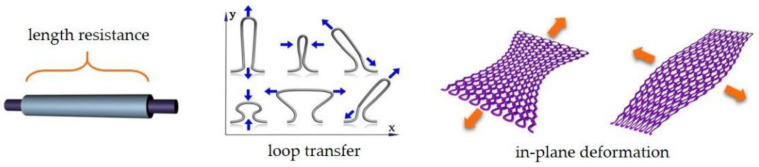
The concepts of length resistance, loop transfer, and in-plane deformation.

This paper proposes two equivalent resistance models of knitted strain sensors under an in-plane deformation. They are macro–micro combined models based on length resistance and a topological equivalent resistance network based on segmented loop structure. In addition, a three-dimensional curved surface equivalent resistance model based on volume resistivity is offered. Comparing the theoretical model and experimental results, it is proven that in-plane and three-dimensional curved surface resistance models can be used to calculate the knitted strain sensor network. Key factors affecting the in-plane knitted strain sensing performance are the length resistance and the loop transfer, as shown in Figure 1. In three-dimensional surface sensing, the key factor influencing the sensing performance is the depth of the surface deformation.

## 2. Materials and Methods

### 2.1. Materials

The knitted strain sensor samples used in the experiments included a commercial silver-coated nylon filament yarn (40 dtex/12 f) purchased from the Tianyin Textile Technology Company (Qingdao, China). The silver-coated nylon yarn’s normalized electrical resistance was 14 Ω/cm, with a resistivity of 0.1 ± 0.05 Ω·m and a tensile strength of 5.6 cN/dtex. Ordinary nylon filament yarn (75 dtex/36 f) and nylon-wrapped spandex yarn (50 dtex spandex filament covered by 20 dtex nylon staple fiber) were purchased from the Kejia Textile Fiber Products Company (Nantong, China). The ordinary nylon yarn was used as received for making a knitted base fabric, and was treated as an insulating material. A single circular knitting (SM8-TOP2 MP2, Pitch 0.907 mm, E28, Diameter 15 inch, Santoni Spa, Italy) machine was used for making conductive knitted fabric. The silver-coated yarn was knitted into the fabric as plating stitches. The specimen’s off-loom course density was 130 wales/5 cm, and its wale density was 85 courses/5 cm. The size of the sensors was 5 cm × 1.5 cm (126 courses × 26 wales).

### 2.2. Sensing Performance Test

**Figure 2:**
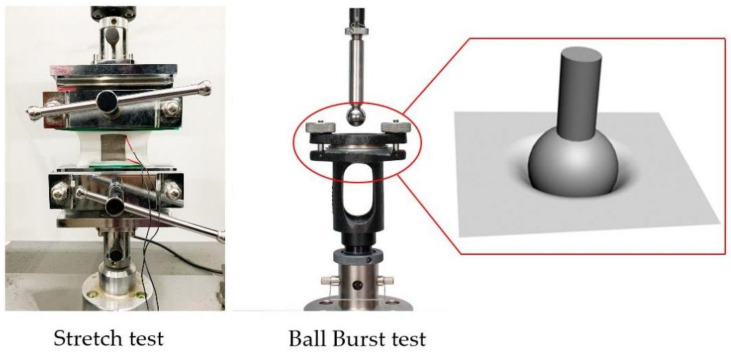
Experimental equipment.

The load and strain of the knitted strain sensor were measured by an Instron Model 5966, in which two pairs of clamps were used to fix the fabric in the wale direction. After selecting this machine, a sensor was fixed in the bottom carrier with a bursting tool, and a ball was installed on the top to perform a curved strain sensing test on the sensor, as shown in Figure 2. The resistance variation of the knitted sensor was tested by a resistance acquisition program using a two-wire method to connect with the sample. This program was a real-time resistance acquisition program specially designed for knitted sensors.

In accordance with the ASTM D5035 standard test method for the breaking force and elongation of textile fabrics, a specimen was stretched in the wale direction. In the experiments, the speed of the clamps was 100 mm/min, the gauge length was 100 mm, and the pre-load was 0.1 N. The relationship between strain and resistance was tested, and repeatability was tested with 2800 stretching cycles at 250 mm/min speed. The in-plane strain-resistance properties were tested by stretching the samples to produce a 100% strain 30 times under the same environment and calculating their resistance ratios, and the testing of the resistance change of the strain sensor was carried out in 2 courses × 27 wales with 50% stretch and calculating the ratios. Three-dimensional surface sensing performance was tested with a set of 400 mm/min for tensile rate and 60% strain.

### 2.3. Theoretical Model

In-plane elongation is generally divided into two categories, course- and wale-stretch elongation [46]. The longitudinal elongation of the knitted fabric is greater than the transverse direction, and the longitudinal direction of the conductive fabric is used as the sensor’s sensing direction.

Alternatively, the three-dimensional curved surface deformation can be approximated as a bursting experimental principle. Through the vertical drop of the sphere, the fabric is required to produce strains in all directions in the three-dimensional space. These two strain methods include the mechanical behavior of the sensor in various spatial directions, which is the basic premise for further research on the in-plane and three-dimensional curved surface strain sensing characteristics of knitted strain sensors.

The change of loop form is one of the main changing characteristics of a knitted strain sensor structure under both in-plane and three-dimensional stretching. The lengths of the loop segments change with loop transmission. When the arc of a parallel and sinker loop is transferred to a leg of loop under the longitudinal tensile, the loop section is transferred to the loop column part. Its tensile resistance deformation process is assumed to be the following steps:

(1) In the unstretched state, fabric loops are tightly abutted, and the resistance of the circuit is low. When subjected to longitudinal stretching, according to the law of resistance as given in Equation (1), the overall circuit resistance increases due to the length change.
  (1) $$R=\frac{\rho L}{S}$$
  where ρ is the proportionality constant and known as the resistivity or the specific resistance of the material of the conductor or substance; L is the length of the substance; S is the cross-sectional area of the substance.
(2) When the force is stretched until loops are separated from each other, the resistance is constant at the maximum value.
(3) When the fabric continues to be stressed, according to the contact resistance [23,24] theory as given in Equation (2), the contact pressure between the loops increases and the resistance decreases.
  (2) $$R_{C}=\frac{\rho }{2}\sqrt{\frac{\pi H}{\mathrm{np}}}$$
  where ρ (${\Omega m}^{-1}$), H (${\mathrm{Nm}}^{-2}$), n, and p (N) are the electrical resistivity, material hardness, number of contact points, and contact pressure between the conductive yarn, respectively.

### 2.4. Model 1: Macro–Micro Equivalent Resistance Models Based on Length Resistance

**Figure 3:**
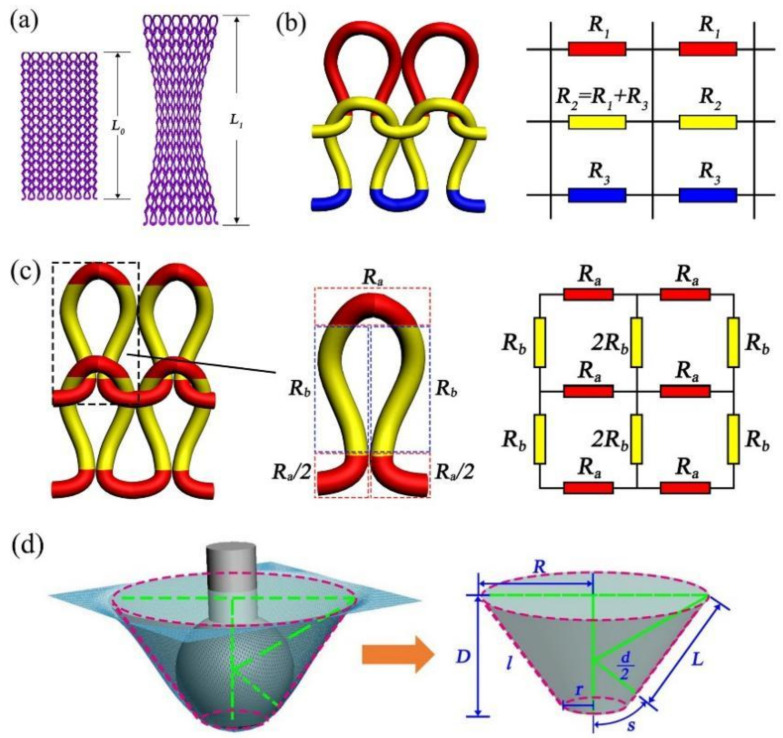
(**a**) macro structure equivalent resistance model; (**b**) micro structure equivalent resistance model; (**c**) topological structure equivalent resistance model; (**d**) three-dimensional surface equivalent resistance model.

From a macro perspective, it was assumed that the conductive area of knitted fabric was a whole yarn, the overall length was $L_{0}$, the cross-sectional area of the fabric was $S_{0}$, and the resistance was $R_{0}$. When the fabric was stretched and deformed, the overall length changed. Therefore, the length, cross-sectional area, and resistance were $L_{1}$, $S_{1}$ and $R_{1}$, respectively, as shown in Figure 3a. Then, from a microscopic point of view, there was resistance on each course of the loop. Taking 2 courses × 2 wales as an example, the arc of a parallel and leg of loop column constituted the first red line of course resistance ($R_{1}$), the sinker loop and the second-course arc of a parallel/leg of loop column constituted a second yellow line of resistance ($R_{2}$), and the second-course sinker loop constituted a third blue line of resistance ($R_{3}$), as shown in Figure 3b. The combination of the red and blue line segments was the yellow loop, and the resistance was Equation (3). According to the 2 courses × 2 wales circuit networks and the series-parallel resistance calculation method, the equivalent resistance equation was Equation (4).

(3) $$R_{2}=R_{1}+R_{3}$$

(4) $$\frac{1}{R_{\left(2,2\right)}}=2\left(\frac{1}{R_{1}}+\frac{1}{R_{2}}+\frac{1}{R_{3}}\right),R_{\left(2,2\right)}=2\left(\frac{R_{1}{}^{2}+R_{3}{}^{2}+3R_{1}R_{3}}{R_{1}{}^{2}R_{3}+R_{3}{}^{2}R_{1}}\right)$$

### 2.5. Model 2: The Equivalent Resistance of the Knitted Sensor Based on the Topology Model

Figure 3c shows the loop structure of the knitted strain sensor, and the equivalent resistance is represented by the topology model. The knitting loop contained by the arc of parallel, sinker loop, and loop column. The arc of parallel had the same resistance as the sinking loop, the arc of parallel resistance was $R_{a}$, the sinking arc was $R_{a}$/2, and the loop column resistance was $R_{b}$. Taking the structure of 2 courses × 2 wales as an example, in the initial state, the conductive knitted fabric was under the influence of spandex, the loops were in close contact, and the loop column and the loop column and the settlement arc and the settlement arc were close to each other. Therefore, the mutual contact resistance of the loop columns was $2R_{b}$, and the mutual contact resistance of the sinking loop was $R_{a}$.

### 2.6. Model 3: The Equivalent Resistance of the Knitted Sensor Based on the Topology Model

Depending on the characteristics of the three-dimensional curved surface strain sensor, the sensors strain surface area change was approximated as a frustum of a cone. As shown in Figure 3d, in the burst test, R was the upper-bottom surface cone radius, r was the bottom surface cone radius, and l was the generatrix of a cone. Therefore, the three-dimensional surface resistance was calculated according to the volume resistivity formula, which refers to material resistance of current per unit volume, and is used to characterize material electrical properties.

(5) $$R_{v}=\frac{\rho D}{S_{v}}$$

where $R_{v}$ is the three-dimensional curved surface deformation resistance value, $S_{v}$ is the curved surface deformation area, D is the sensor’s burst depth, and ρ is volume resistivity in Ω/cm.

## 3. Results

### 3.1. Calculation of the Macro–Micro Equivalent Resistance Model Based on Length Resistance

Calculated from the macrostructure at a relaxed state (according to the law of resistance), when the fabric is in its unstretched state, its length, cross-sectional area, and resistance are $L_{0}$, $S_{0}$, and $R_{0}$, respectively. The strain rate is ε for the in-plane stretching. Its length, cross-sectional area, and resistance are $L_{1}$, $S_{1}$, and $R_{1}$ after the in-plane stretching, as shown in Equation (6). Therefore, the theoretical model of the equivalent resistance ratio of the in-plane strain can be deduced from Equation (7). The calculation resistance ratio $R^{\prime }/R$ is assumed to be $y_{1}$, where the relationship between $y_{1}$ and strain ε is:

(6) $$\left\{\begin{array}{l} R_{0}=\frac{{\rho L}_{0}}{S_{0}}=\frac{{\rho L}_{0}}{{\pi r}^{2}}\;\\ L_{1}=\varepsilon L_{0}+L_{0}\\ S_{1}=\pi {\left(\frac{r}{\varepsilon +1}\right)}^{2}\\ R_{1}=\frac{\rho \left(\varepsilon +1\right)L_{0}}{\pi {\left(\frac{r}{\varepsilon +1}\right)}^{2}}=\frac{{\rho L}_{0}{\left(\varepsilon +1\right)}^{3}}{{\pi r}^{2}}\\ \frac{R_{1}}{R_{0}}=\varepsilon^{3}+3\varepsilon^{2}+3\varepsilon +1 \end{array}\right.$$

(7) $$y_{1}=\varepsilon^{3}+3\varepsilon^{2}+3\varepsilon +1$$

**Figure 4:**
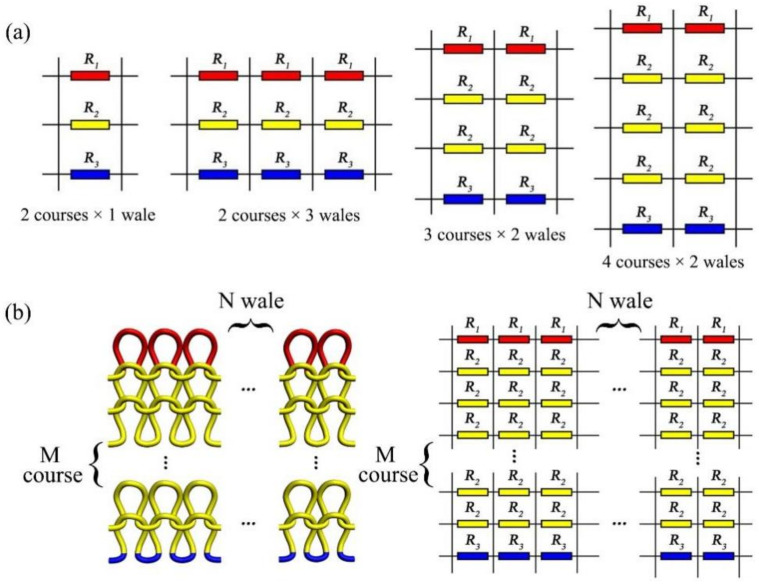
(**a**) 2 courses × 1 wale, 2 courses × 3 wales, 3 courses × 2 wales, and 4 courses × 2 wales equivalent circuits; (**b**) The equivalent circuit of M course × N wale.

Calculated from the microstructure, according to the 2 courses × 2 wales equivalent circuit and by using the same method, Equation (8) shows that in the relationship among the equivalent resistances of 2 courses × 1 wale, 2 courses × 3 wales, 3 courses × 2 wales, and 4 courses × 2 wales, the series-parallel circuit’s connection of equivalent resistance of knitting sensor exists in course and wale direction. Therefore, the M course × N wale equivalent resistance of the knitted sensor can be deduced from Equation (9); it should be noted that M and N refer to the number of loop courses and wales in the sensing area, respectively, as shown in Figure 4.

(8) $$\left\{\begin{array}{l} \frac{1}{R_{\left(2,1\right)}}=\frac{1}{R_{1}}+\frac{1}{R_{2}}+\frac{1}{R_{3}},R_{\left(2,1\right)}=\frac{R_{1}{}^{2}+R_{3}{}^{2}+3R_{1}R_{3}}{R_{1}{}^{2}R_{3}+R_{3}{}^{2}R_{1}}\\ \frac{1}{R_{\left(2,2\right)}}=2\left(\frac{1}{R_{1}}+\frac{1}{R_{2}}+\frac{1}{R_{3}}\right),R_{\left(2,2\right)}=2\left(\frac{R_{1}{}^{2}+R_{3}{}^{2}+3R_{1}R_{3}}{R_{1}{}^{2}R_{3}+R_{3}{}^{2}R_{1}}\right)\\ \frac{1}{R_{\left(2,3\right)}}=3\left(\frac{1}{R_{1}}+\frac{1}{R_{2}}+\frac{1}{R_{3}}\right),R_{\left(2,3\right)}=3\left(\frac{R_{1}{}^{2}+R_{3}{}^{2}+3R_{1}R_{3}}{R_{1}{}^{2}R_{3}+R_{3}{}^{2}R_{1}}\right)\\ \frac{1}{R_{\left(3,2\right)}}=2\left(\frac{1}{R_{1}}+\frac{1}{R_{2}}+\frac{1}{R_{2}}+\frac{1}{R_{3}}\right),R_{\left(3,2\right)}=2\left(\frac{R_{1}{}^{2}+R_{3}{}^{2}+4R_{1}R_{3}}{R_{1}{}^{2}R_{3}+R_{3}{}^{2}R_{1}}\right)\\ \frac{1}{R_{\left(4,2\right)}}=4\left(\frac{1}{R_{1}}+\frac{1}{R_{2}}+\frac{1}{R_{2}}+\frac{1}{R_{2}}+\frac{1}{R_{3}}\right),R_{\left(4,2\right)}=4\left(\frac{R_{1}{}^{2}+R_{3}{}^{2}+5R_{1}R_{3}}{R_{1}{}^{2}R_{3}+R_{3}{}^{2}R_{1}}\right) \end{array}\right.$$

(9) $$\frac{1}{R_{\left(M,N\right)}}=N\left(\frac{1}{R_{1}}+\left(M-1\right)\frac{1}{R_{2}}+\frac{1}{R_{3}}\right),R_{\left(M,N\right)}=N\left(\frac{R_{1}{}^{2}+R_{3}{}^{2}+\left(M+1\right)R_{1}R_{3}}{R_{1}{}^{2}R_{3}+R_{3}{}^{2}R_{1}}\right)(M>1)$$

When knitted fabrics are in-plane stretched an extending of each loop and yarn segment occurs, transferring among the arc of parallel, leg and sinker loop. Then, the adjacent loops become tightly interlinked with each other. Due to the large strain caused by the range of extension, the following assumptions are considered to simplify the structure of knitted loop under a large strain. Assuming that the resistance changes of $R_{1}$, $R_{2}$, and $R_{3}$ are approximately equal (for the simple calculation, the three resistances are approximately equal to $R_{2}$), then the total resistance (R) of the network and the resistance after stretching ($R^{\prime }$) can be calculated as in Equation (10). The equivalent resistance ratio $R^{\prime }/R$ is assumed to be $y_{2}$, where the relationship between $y_{2}$, strain ε, course, and wale is presented as Equation (11).

(10) $$\left\{\begin{array}{l} \frac{1}{R}=\frac{1}{N}\left(\frac{1}{R_{2}}+\frac{1}{R_{2}}+\cdots +\frac{M}{R_{2}}\right)\\ R=N\frac{R_{2}}{M+1}=N\frac{\frac{\rho L}{S}}{M+1}\\ L^{\prime }=\left(\varepsilon +1\right)L,\;S^{\prime }=\pi {\left(\frac{r}{\varepsilon +1}\right)}^{2}\\ R^{\prime }=\frac{\rho L^{\prime }}{S^{\prime }}=\frac{\rho 2\left(\varepsilon +1\right)L}{\pi {\left(\frac{r}{\varepsilon +1}\right)}^{2}}\\ \frac{R^{\prime }}{R}=N\frac{2{\left(\varepsilon +1\right)}^{3}}{M+1} \end{array}\right.$$

(11) $$y_{2}=N\frac{2{\left(\varepsilon +1\right)}^{3}}{M+1}$$

### 3.2. Calculation of the Equivalent Resistance Based on the Topology Model

According to Kirchhoff’s current and voltage law, the equivalent resistance of the topology model and the corresponding equations can be calculated [47]. The total current (I_1_) in the circuit can be calculated by Matlab, and the equivalent resistance (R) of the knitted strain sensor can be defined as Equation (12), where V is the total voltage that the circuit loads; (I_1_) is the total current in the circuit.

(12) $$R=\frac{V}{I_{1}}$$

**Figure 5:**
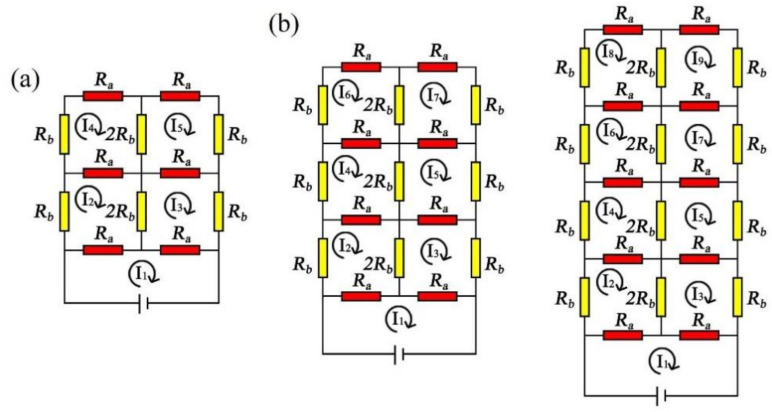
(**a**) 2 courses × 2 wales loop circuit; (**b**) 3 courses × 2 wales and 4 courses × 2 wales loop circuits.

Based on the geometric structure of the knitting loop, the knitting strain sensor starts with 2 courses × 2 wales and extends to M course × N wale. Kirchhoff’s current and voltage law is used to calculate the total circuit $I_{1}$ of the knitted fabric circuit. Figure 5a is a loop circuit with 2 courses × 2 wales. The knitting sensor is connected to the power supply at both ends, and its voltage is V. The circuit equations calculated by Kirchhoff’s current and voltage law are shown in Equation (13), and this circuit contains 5 loop currents. Solving the above equation with Matlab comprehensively calculates the equivalent resistance of the 2 courses × 2 wales loop circuits as Equation (14).

The same method can be used to solve for the equivalent resistances of the 3 courses × 2 wales loop circuits and 4 courses × 2 wales loop circuits, with the circuit equation set calculated by Kirchhoff’s current and voltage laws, as shown in Figure 5b and the Equations (15) and (16). The 3 courses × 2 wales circuits contain seven loop circuits, and the 4 courses × 2 wales circuits contain nine loop currents.

(13) $$\left\{\begin{array}{l} 2I_{1}R_{a}-I_{2}R_{a}-I_{3}R_{a}=V\\ I_{2}\left(2R_{a}+3R_{b}\right)-I_{1}R_{a}-I_{4}R_{a}-2I_{3}R_{b}=0\\ I_{3}\left(2R_{a}+3R_{b}\right)-I_{1}R_{a}-I_{5}R_{a}-2I_{2}R_{b}=0\\ I_{4}\left(2R_{a}+3R_{b}\right)-2I_{5}R_{b}-I_{2}R_{a}=0\\ I_{5}\left(2R_{a}+3R_{b}\right)-I_{3}R_{a}-2I_{4}R_{b}=0 \end{array}\right.$$

(14) $$R_{\left(2,2\right)}=\frac{2R_{a}\left(R_{a}{}^{2}+3R_{a}R_{b}+R_{b}{}^{2}\right)}{3R_{a}{}^{2}+R_{b}{}^{2}+4R_{a}R_{b}}$$

(15) $$\left\{\begin{array}{l} 2I_{1}R_{a}-I_{2}R_{a}-I_{3}R_{a}=V\\ I_{2}\left(2R_{a}+3R_{b}\right)-I_{1}R_{a}-I_{4}R_{a}-2I_{3}R_{b}=0\\ I_{3}\left(2R_{a}+3R_{b}\right)-I_{1}R_{a}-I_{5}R_{a}-2I_{2}R_{b}=0\\ I_{4}\left(2R_{a}+3R_{b}\right)-I_{2}R_{a}-I_{6}R_{a}-2I_{5}R_{b}=0\\ I_{5}\left(2R_{a}+3R_{b}\right)-I_{3}R_{a}-I_{7}R_{a}-2I_{4}R_{b}=0\\ I_{6}\left(2R_{a}+3R_{b}\right)-I_{4}R_{a}-2I_{7}R_{b}=0\\ I_{7}\left(2R_{a}+3R_{b}\right)-I_{5}R_{a}-2I_{6}R_{b}=0 \end{array}\right.$$

(16) $$\left\{\begin{array}{l} 2I_{1}R_{a}-I_{2}R_{a}-I_{3}R_{a}=V\\ I_{2}\left(2R_{a}+3R_{b}\right)-I_{1}R_{a}-I_{4}R_{a}-2I_{3}R_{b}=0\\ I_{3}\left(2R_{a}+3R_{b}\right)-I_{1}R_{a}-I_{5}R_{a}-2I_{2}R_{b}=0\\ I_{4}\left(2R_{a}+3R_{b}\right)-I_{2}R_{a}-I_{6}R_{a}-2I_{5}R_{b}=0\\ I_{5}\left(2R_{a}+3R_{b}\right)-I_{3}R_{a}-I_{7}R_{a}-2I_{4}R_{b}=0\\ I_{6}\left(2R_{a}+3R_{b}\right)-I_{4}R_{a}-I_{8}R_{a}-2I_{7}R_{b}=0\\ I_{7}\left(2R_{a}+3R_{b}\right)-I_{5}R_{a}-I_{9}R_{a}-2I_{6}R_{b}=0\\ I_{8}\left(2R_{a}+3R_{b}\right)-I_{6}R_{a}-2I_{9}R_{b}=0\\ I_{9}\left(2R_{a}+3R_{b}\right)-I_{7}R_{a}-2I_{8}R_{b}=0 \end{array}\right.$$

The equivalent resistance of the 3 courses × 2 wales and 4 courses × 2 wales loop circuits can be comprehensively calculated by solving the above equation with Matlab, as in the Equations (17) and (18). The mathematical transformation of the equivalent resistance Equations (14), (17) and (18) for 2 courses × 2 wales, 3 courses × 2 wales, and 4 courses × 2 wales results in the following formulas being obtained:

(17) $$R_{\left(3,2\right)}=\frac{2R_{a}\left(R_{a}{}^{3}+6R_{a}{}^{2}R_{b}+5R_{a}R_{b}{}^{2}+R_{b}{}^{3}\right)}{4R_{a}{}^{3}+R_{b}{}^{3}+10R_{a}{}^{2}R_{b}+6R_{a}R_{b}{}^{2}}$$

(18) $$R_{\left(4,2\right)}=\frac{2R_{a}\left(R_{a}{}^{4}+10R_{a}{}^{3}R_{b}+15R_{a}{}^{2}R_{b}{}^{2}+7R_{a}R_{b}{}^{3}+R_{b}{}^{4}\right)}{5R_{a}{}^{4}+R_{b}{}^{4}+20R_{a}{}^{3}R_{b}+21R_{a}{}^{2}R_{b}{}^{2}+8R_{a}R_{b}{}^{3}}$$

(19) $$\left\{\begin{array}{l} R_{\left(2,2\right)}=\frac{2R_{a}\left[{\left(R_{a}+R_{b}\right)}^{2}+R_{a}R_{b}\right]}{{\left(R_{a}+R_{b}\right)}^{2}+2R_{a}\left(R_{a}+R_{b}\right)}=\left(R_{a}+R_{b}\right)-\frac{R_{a}{}^{3}+R_{a}{}^{2}R_{b}+3R_{a}R_{b}{}^{2}+R_{b}{}^{3}}{3R_{a}{}^{2}+4R_{a}R_{b}+R_{b}{}^{2}}\\ R_{\left(3,2\right)}=\frac{2}{3}\left(R_{a}+R_{b}\right)-\frac{2(R_{a}{}^{4}-4R_{a}{}^{3}R_{b}+R_{a}{}^{2}R_{b}{}^{2}+4R_{a}R_{b}{}^{3}+R_{b}{}^{4})}{3(4R_{a}{}^{3}+10R_{a}{}^{2}R_{b}+6R_{a}R_{b}{}^{2}+R_{b}{}^{3})}\\ R_{\left(4,2\right)}=\frac{1}{2}\left(R_{a}+R_{b}\right)-\frac{R_{a}{}^{5}-15R_{a}{}^{4}R_{b}-19R_{a}{}^{3}R_{b}{}^{2}+R_{a}{}^{2}R_{b}{}^{3}+5R_{a}R_{b}{}^{4}+R_{b}{}^{5}}{2\left(5R_{a}{}^{4}+20R_{a}{}^{3}R_{b}+21R_{a}{}^{2}R_{b}{}^{2}+8R_{a}R_{b}{}^{3}+R_{b}{}^{4}\right)} \end{array}\right.$$

It is impossible to find the rule of M course × 2 wales from this expression. Therefore, if Equation (19) is factorized, the denominator of the last term in the equation is much larger than the numerator. When there are more courses, the last term in the three expressions is approximately zero and can be ignored. Thus, the following relationship is obtained:

(20) $$\left\{\begin{array}{l} R_{\left(2,2\right)}\approx \left(R_{a}+R_{b}\right)\\ R_{\left(3,2\right)}\approx \frac{2}{3}\left(R_{a}+R_{b}\right)\\ R_{\left(4,2\right)}\approx \frac{1}{2}\left(R_{a}+R_{b}\right)\\ R_{\left(M,2\right)}\approx \frac{2}{M}\left(R_{a}+R_{b}\right) \end{array}\right.$$

**Figure 6:**
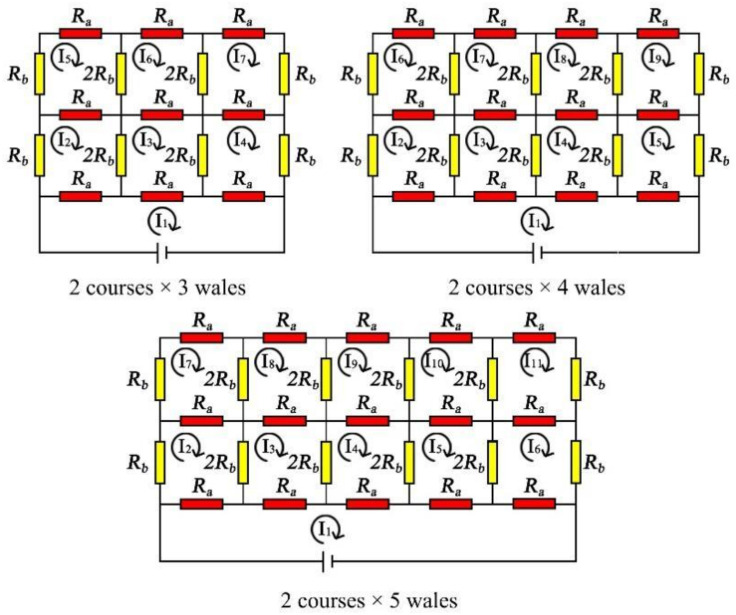
The equivalent resistances of the circuits with 2 courses × 3 wales, 2 courses × 4 wales, and 2 courses × 5 wales.

It is inferred that along the wale direction of the knitted sensor, the equivalent resistance decreases with the increase of courses. The circuit is a parallel circuit, and the equivalent resistance expression of M course × 2 wales is Equation (21). Figure 6 shows the equivalent resistances of the circuits with 2 courses × 3 wales, 2 courses × 4 wales, and 2 courses × 5 wales. The equivalent resistances are calculated by the same method, and the equations obtained are shown in Equations (22)–(24).

(21) $$R_{\left(M,2\right)}=\frac{2}{M}\left(R_{a}+R_{b}\right)$$

(22) $$\left\{\begin{array}{l} 3I_{1}R_{a}-I_{2}R_{a}-I_{3}R_{a}-I_{4}R_{a}=V\\ I_{2}\left(2R_{a}+3R_{b}\right)-I_{1}R_{a}-2I_{3}R_{b}-I_{5}R_{a}=0\\ I_{3}\left(2R_{a}+4R_{b}\right)-I_{1}R_{a}-2I_{2}R_{b}-I_{6}R_{a}-2I_{4}R_{b}=0\\ I_{4}\left(2R_{a}+3R_{b}\right)-2I_{3}R_{b}-I_{1}R_{a}-I_{7}R_{a}=0\\ I_{5}\left(2R_{a}+3R_{b}\right)-I_{2}R_{a}-2I_{6}R_{b}=0\\ I_{6}\left(2R_{a}+4R_{b}\right)-I_{3}R_{a}-2I_{5}R_{b}-2I_{7}R_{b}=0\\ I_{7}\left(2R_{a}+3R_{b}\right)-I_{4}R_{a}-2I_{6}R_{b}=0 \end{array}\right.$$

(23) $$\left\{\begin{array}{l} 4I_{1}R_{a}-I_{2}R_{a}-I_{3}R_{a}-I_{4}R_{a}-I_{5}R_{a}=V\\ I_{2}\left(2R_{a}+3R_{b}\right)-I_{1}R_{a}-2I_{3}R_{b}-I_{6}R_{a}=0\\ I_{3}\left(2R_{a}+4R_{b}\right)-I_{1}R_{a}-2I_{2}R_{b}-I_{7}R_{a}-2I_{4}R_{b}=0\\ I_{4}\left(2R_{a}+4R_{b}\right)-2I_{3}R_{b}-2I_{5}R_{b}-I_{1}R_{a}-I_{8}R_{a}=0\\ I_{5}\left(2R_{a}+3R_{b}\right)-I_{1}R_{a}-I_{9}R_{a}-2I_{4}R_{b}=0\\ I_{6}\left(2R_{a}+3R_{b}\right)-I_{2}R_{a}-2I_{7}R_{b}=0\\ I_{7}\left(2R_{a}+4R_{b}\right)-I_{3}R_{a}-2I_{6}R_{b}-2I_{8}R_{b}=0\\ I_{8}\left(2R_{a}+4R_{b}\right)-I_{4}R_{a}-2I_{7}R_{b}-2I_{9}R_{b}=0\\ I_{9}\left(2R_{a}+3R_{b}\right)-I_{5}R_{a}-2I_{8}R_{b}=0 \end{array}\right.$$

(24) $$\left\{\begin{array}{l} 5I_{1}R_{a}-I_{2}R_{a}-I_{3}R_{a}-I_{4}R_{a}-I_{5}R_{a}-I_{6}R_{a}=V\\ I_{2}\left(2R_{a}+3R_{b}\right)-I_{1}R_{a}-I_{3}2R_{b}-I_{7}R_{a}=0\\ I_{3}\left(2R_{a}+4R_{b}\right)-I_{1}R_{a}-I_{8}R_{a}-I_{2}2R_{b}-I_{4}2R_{b}=0\\ I_{4}\left(2R_{a}+4R_{b}\right)-I_{1}R_{a}-I_{9}R_{a}-I_{3}2R_{b}-I_{5}2R_{b}=0\\ I_{5}\left(2R_{a}+4R_{b}\right)-I_{1}R_{a}-I_{10}R_{a}-I_{4}2R_{b}-I_{6}2R_{b}=0\\ I_{6}\left(2R_{a}+3R_{b}\right)-I_{1}R_{a}-I_{11}R_{a}-I_{5}2R_{b}=0\\ I_{7}\left(2R_{a}+3R_{b}\right)-I_{2}R_{a}-I_{8}2R_{b}=0\\ I_{8}\left(2R_{a}+4R_{b}\right)-I_{3}R_{a}-I_{7}2R_{b}-I_{9}2R_{b}=0\\ I_{9}\left(2R_{a}+4R_{b}\right)-I_{4}R_{a}-I_{8}2R_{b}-I_{10}2R_{b}=0\\ I_{10}\left(2R_{a}+4R_{b}\right)-I_{5}R_{a}-I_{9}2R_{b}-I_{11}2R_{b}=0\\ I_{11}\left(2R_{a}+3R_{b}\right)-I_{6}R_{a}-I_{10}2R_{b}=0 \end{array}\right.$$

**Figure 7:**
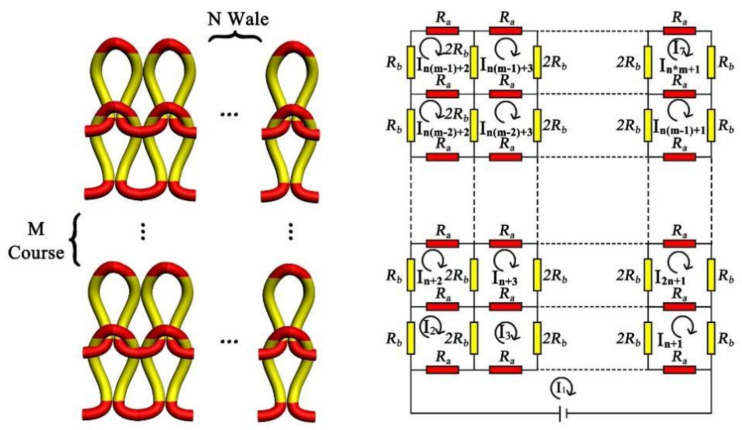
The equivalent circuit of the knitted strain sensor with M course × N wale.

Through the resistance formulas of 2 courses × 3 wales, 2 courses × 4 wales, and 2 courses × 5 wales (Equation (25)), the resistance expression law of 2 courses × N wale cannot be directly obtained, so the equivalent resistance of the knitted sensor topological model can instead be obtained through the corresponding equations of M course × N wale, as in Equation (26), and as shown in Figure 7 (the equivalent circuit of the knitted strain sensor with M course × N wale).

(25) $$\left\{\begin{array}{l} R_{\left(2,3\right)}=\frac{R_{a}\left(9R_{a}{}^{4}+94R_{a}{}^{3}R_{b}+271R_{a}{}^{2}R_{b}{}^{2}+260R_{a}R_{b}{}^{3}+48R_{b}{}^{4}\right)}{9R_{a}{}^{4}+16R_{b}{}^{4}+84R_{a}{}^{3}R_{b}+187R_{a}{}^{2}R_{b}{}^{2}+112R_{a}R_{b}{}^{3}}\\ R_{\left(2,4\right)}=\frac{2R_{a}\left(6R_{a}{}^{4}+45R_{a}{}^{3}R_{b}+92R_{a}{}^{2}R_{b}{}^{2}+62R_{a}R_{b}{}^{3}+8R_{b}{}^{4}\right)}{9R_{a}{}^{4}+4R_{b}{}^{4}+60R_{a}{}^{3}R_{b}+95R_{a}{}^{2}R_{b}{}^{2}+40R_{a}R_{b}{}^{3}}\\ R_{\left(2,5\right)}=\\ \frac{R_{a}\left(45R_{a}{}^{6}+690R_{a}{}^{5}R_{b}+3627R_{a}{}^{4}R_{b}{}^{2}+8348R_{a}{}^{3}R_{b}{}^{3}+8592R_{a}{}^{2}R_{b}{}^{4}+3520R_{a}R_{b}{}^{5}+320R_{b}{}^{6}\right)}{27R_{a}{}^{4}+64R_{b}{}^{4}+396R_{a}{}^{6}R_{b}+1929R_{a}{}^{4}R_{b}{}^{2}+3920R_{a}{}^{3}R_{b}{}^{3}+3232R_{a}{}^{2}R_{b}{}^{4}+896R_{a}R_{b}{}^{5}} \end{array}\right.$$

(26) $$\left\{\begin{array}{l} \left(N+1\right)I_{1}R_{a}-I_{2}R_{a}-I_{3}R_{a}-I_{4}R_{a}-\cdots -I_{N+1}R_{a}=V\\ I_{2}\left(2R_{a}+3R_{b}\right)-I_{1}R_{a}-I_{3}2R_{b}-I_{N+2}R_{a}=0\\ I_{3}\left(2R_{a}+4R_{b}\right)-I_{1}R_{a}-I_{2}2R_{b}-I_{4}2R_{b}-I_{N+3}R_{a}=0\\ \vdots \\ I_{N+1}\left(2R_{a}+3R_{b}\right)-I_{1}R_{a}-I_{2N+1}R_{a}-I_{N}2R_{b}=0\\ I_{N+2}\left(2R_{a}+3R_{b}\right)-I_{2}R_{a}-I_{2N+2}R_{a}-I_{N+3}2R_{b}=0\\ I_{N+3}\left(2R_{a}+4R_{b}\right)-I_{3}R_{a}-I_{2N+3}R_{a}-I_{N+2}2R_{b}-I_{N+4}2R_{b}=0\\ \vdots \\ I_{2N+1}\left(2R_{a}+3R_{b}\right)-I_{N+1}R_{a}-I_{3N+1}R_{a}-I_{2N}2R_{b}=0\\ \vdots \\ I_{\left(M-2\right)N+2}\left(2R_{a}+3R_{b}\right)-I_{\left(M-1\right)N+2}R_{a}-I_{\left(M-3\right)N+2}R_{a}-I_{\left(M-2\right)N+3}2R_{b}=0\\ I_{\left(M-2\right)N+3}\left(2R_{a}+4R_{b}\right)-I_{\left(M-1\right)N+3}R_{a}-I_{\left(M-3\right)N+3}R_{a}-I_{\left(M-2\right)N+2}2R_{b}-I_{\left(M-2\right)N+4}2R_{b}=0\\ \vdots \\ I_{\left(M-1\right)N+1}\left(2R_{a}+3R_{b}\right)-I_{\mathrm{MN}+1}R_{a}-I_{\left(M-2\right)N+1}R_{a}-I_{\left(M-1\right)N}2R_{b}=0\\ I_{\left(M-1\right)N+2}\left(2R_{a}+3R_{b}\right)-I_{\left(M-2\right)N+2}R_{a}-I_{\left(M-1\right)N+3}2R_{b}=0\\ I_{\left(M-1\right)N+3}\left(2R_{a}+4R_{b}\right)-I_{\left(M-2\right)N+3}R_{a}-I_{\left(M-1\right)N+2}2R_{b}-I_{\left(M-1\right)N+4}2R_{b}=0\\ \vdots \\ I_{\mathrm{MN}+1}\left(2R_{a}+3R_{b}\right)-I_{\left(M-1\right)N+1}R_{a}-I_{\mathrm{MN}}2R_{b}=0 \end{array}\right.$$

According to the M course × N wale topology model equations on loop transfer and length resistance (take 2 courses × 2 wales as an example to calculate the equivalent resistance ratio during longitudinal strain), $R_{a}{}^{\prime }$, $R_{b}{}^{\prime }$, and the equivalent resistance ratio ${R^{\prime }}_{\left(2,2\right)}/R_{\left(2,2\right)}$ of 2 courses × 2 wales can be calculated, as shown in Equation (27).

(27) $$\left\{\begin{array}{l} R_{\left(2,2\right)}=\frac{2R_{a}\left(R_{a}{}^{2}+3R_{a}R_{b}+R_{b}{}^{2}\right)}{3R_{a}{}^{2}+R_{b}{}^{2}+4R_{a}R_{b}}\\ R_{a}^{\prime }=R_{a}\left(1-\frac{\varepsilon }{2}\right)\\ R_{b}^{\prime }=R_{b}\left(1+\varepsilon \right)\\ R_{\left(2,2\right)}^{\prime }=\frac{2R_{a}\left(1-\frac{\varepsilon }{2}\right)\left({\left[R_{a}\left(1-\frac{\varepsilon }{2}\right)\right]}^{2}+3R_{a}\left(1-\frac{\varepsilon }{2}\right)R_{b}\left(1+\varepsilon \right)+{\left[R_{b}\left(1+\varepsilon \right)\right]}^{2}\right)}{3{\left[R_{a}\left(1-\frac{\varepsilon }{2}\right)\right]}^{2}+{\left[R_{b}\left(1+\varepsilon \right)\right]}^{2}+4R_{a}\left(1-\frac{\varepsilon }{2}\right)R_{b}\left(1+\varepsilon \right)}\\ \frac{{R^{\prime }}_{\left(2,2\right)}}{R_{\left(2,2\right)}}=\frac{\left(3R_{a}{}^{2}+4R_{a}R_{b}+R_{b}{}^{2}\right)\left[2R_{a}{}^{3}{\left(\frac{\varepsilon }{2}-1\right)}^{3}-R_{b}{}^{2}{\left(1+\varepsilon \right)}^{2}+3R_{a}R_{b}\left(\frac{\varepsilon }{2}-1\right)\left(1+\varepsilon \right)\right]}{2R_{a}\left[R_{b}{}^{2}{\left(1+\varepsilon \right)}^{2}+3R_{a}{}^{2}{\left(\frac{\varepsilon }{2}-1\right)}^{2}-4R_{a}R_{b}\left(\frac{\varepsilon }{2}-1\right)\left(1+\varepsilon \right)\right]\left(R_{a}{}^{2}+3R_{a}R_{b}+R_{b}{}^{2}\right)} \end{array}\right.$$

### 3.3. Calculation of the Equivalent Resistance Model Based on the Three-Dimensional Curved Surface Strain

According to the change of the sensor surface area, the relationship between the burst depth (D) and curved surface strain is established. The volume resistivity formula is used to calculate the resistance ($R_{v}$) before breaking and the equivalent resistance ($R_{v}^{\prime }$) after the curved surface strain. The ratio of surface strain resistance is derived as shown in Equation (28). To avoid the burst depth of the formula being zero in an initial state, the burst depth (D) in the initial state is set to 1 mm, and bottom surface cone radius (r) is set to one-half of a stainless steel radius. Therefore, the resistance change ratio of the curved surface strain is defined as $y_{v}$, and the relationship between $y_{v}$ and the burst depth (D) is expressed as Equation (29).

(28) $$\left\{\begin{array}{l} R_{v}=\frac{\rho D}{S_{v}}\\ R_{v}^{\prime }=\frac{\rho D^{\prime }}{S_{v}^{\prime }}=\frac{\rho D^{\prime }}{\pi l^{\prime }\left(R+r\right)+{\pi r}^{2}}\\ l^{\prime }=\sqrt{{\left(R-r\right)}^{2}+D^{\prime }}\\ \frac{R_{v}^{\prime }}{R_{v}}=\frac{S_{v}D^{\prime }}{\pi l^{\prime }D\left(R+r\right)+{\pi r}^{2}D}=\frac{7.5D^{\prime }}{0.88705\sqrt{{D^{\prime }}^{2}-2.5D^{\prime }+4.84}+0.12265625} \end{array}\right.$$

(29) $$y_{v}=\frac{7.5D}{0.88705\sqrt{D^{2}-2.5D+4.84}+0.12265625}$$

### 3.4. Fabric Cycle Test Results under In-Plane Stretching

**Figure 8:**
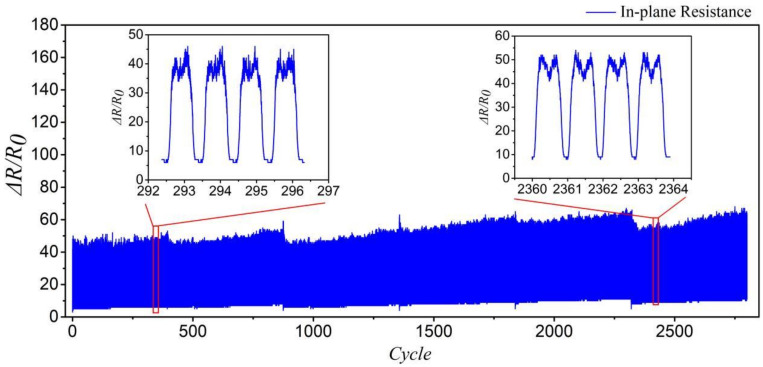
In-plane cycle stretching experiment.

The initial strain responsiveness was tested by stretching the samples to produce a 100% strain at a speed of 300 mm/min. Following 2800 occurrences of in-plane stretching, 50 groups of cycle data were extracted according to the cyclic stretching data for the analysis of variance, as shown in Figure 8. The results show that the *p*-value of the in-plane stretching is 0.955 > 0.05, as shown in Table 1, indicating that the extracted actual resistance data have no significant positive influence on all cycle data. It proved that an equivalent resistance model established for the same knitted sensor is feasible.

### 3.5. Comparison of the Experimental Data and the Equivalent Resistance Model Calculation Results during In-Plane Stretching

**Figure 9:**
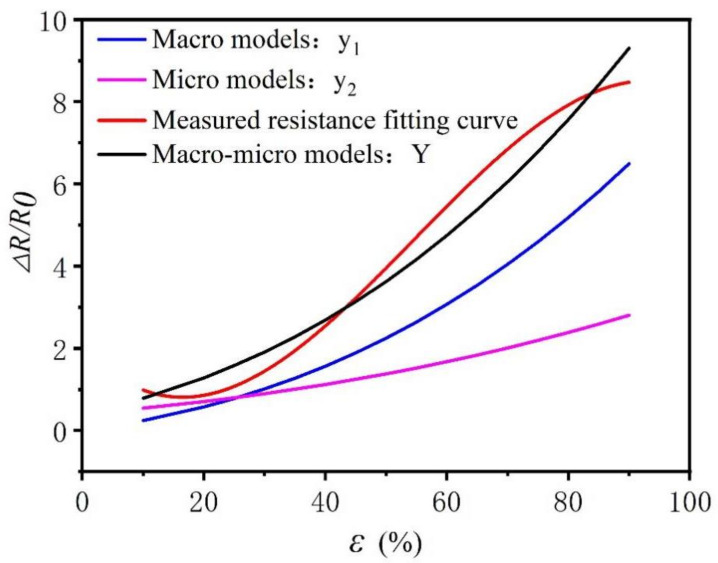
Comparison of the actual resistance and the in-plane tensile equivalent resistance model calculation results.

The equivalent resistance model during in-plane stretching is composed of macro–micro models; that is, the combination of the Equations (7) and (11). Therefore, the in-plane tensile equivalent resistance model Equation (30) can be derived. Figure 9 illustrates the simulation results of the equivalent resistance and the experimental data of the in-plane stretching, where the two curves Y and y are close to each other (within acceptable accuracy), and further analyzes the fit of the two curves. It can be seen from Table 2 that the $R^{2}$ value of the model is 0.975, meaning that the in-plane strain fitting resistance can explain 97.5% of the change of real resistance. When the *F* test was performed on the model, it was found that the *F* test (F=39060.82, p=0.00<0.05) was applied to the model, which indicated that the in-plane fitting resistance curve will have a significant positive impact on a real resistance curve. The equivalent resistance theoretical model of in-plane tensile properties can be used to predict the same knitted fabric resistance according to the given strain, and the same method can be used in future studies to predict the deformation of the fabric based on the actual resistance obtained.

(30) $$Y=y_{1}+y_{2}=\varepsilon^{3}+3\varepsilon^{2}+3\varepsilon +\frac{2n{\left(\varepsilon +1\right)}^{3}}{m+1}+1$$

### 3.6. Comparison of the In-Plane Tensile Test Results and the Equivalent Resistance Calculation Results of the Topology Model

**Figure 10:**
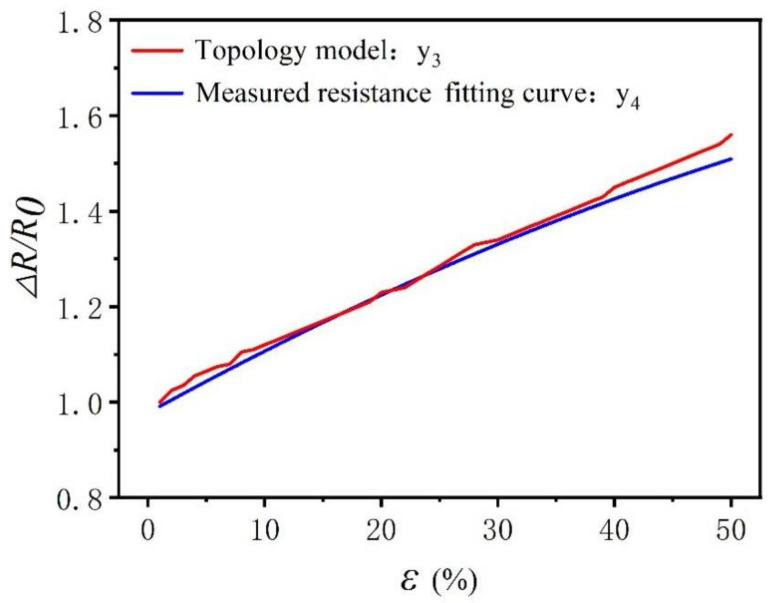
Comparison of actual resistance and topology equivalent resistance model calculation results.

Comparing the simulation results of the knitting sensor topology resistance model with the results of longitudinal stretching shows that the two curves are close to each other within acceptable accuracy, further verifying the fit of the two curves, as shown in Figure 10. It can be seen from Table 3 that the $R^{2}$ value of the model is 0.99, meaning that the topology model fitting resistance can explain 99% of the change of real resistance. When the *F* test was performed on the model, it was found that the *F* test (*F* = 43,544.39, *p* = 0.00<0.05) was applied to the model, which indicated that the topology model fitting the resistance curve will have a significant positive impact on the real resistance curve. The equivalent resistance theoretical model of the topology model can be used to predict the same knitted fabric resistance according to a given strain, and the same method can be used in future studies to predict the deformation of the fabric based on the actual resistance obtained.

Comparing the two equivalent resistance models, the macro–micro model establishes the equivalent resistance of the knitted sensor from two perspectives. When the selected strain sensor is larger in size, the macro–micro model can be used to calculate the equivalent resistance more easily and quickly, and the calculation efficiency is higher. The topology equivalent resistance model comprehensively considers the loop transfer and length resistance. When selecting a smaller sensor application, it is more accurate to use the topology resistance model to calculate the equivalent resistance. Compared with the macro–micro models, the topological model has a higher resistance prediction accuracy, but the calculation is more complicated.

### 3.7. Comparison of Three-Dimensional Strain Equivalent Resistance Model and Test Results

Equation (29) demonstrates a proportional relationship between burst depth and surface strain. Therefore, the relationship between the equivalent resistance ratio and the curved surface strain can be established, as shown in Figure 9. The red curve in Figure 9 aligns with the smooth curve of the burst test data, and the black curve is the predicted result of the equivalent resistance model.

From the analysis in Figure 9, two curves are close to each other within acceptable accuracy, which further verifies the fit of two curves, as shown in Table 4. The fitted resistance is used as an independent variable and actual resistance is used as the dependent variable for linear regression analysis. The accuracy of the fit $R^{2}$ is 0.867, which means that the fitted resistance can explain 86.7% of the actual resistance. The model passes the *F* test (*F* = 366274.180, *p* = 0.000 < 0.05), which means that the fitted resistance will affect the actual resistance. The specific analysis shows that the regression coefficient value of the fitting resistance is 0.888 (*t* = 605.206, *p* = 0.000 < 0.01), which means that the fitting resistance will have a significant positive influence on the actual resistance. Therefore, a three-dimensional curved surface equivalent resistance model can be used to predict the curved surface resistance change.

**Figure 11:**
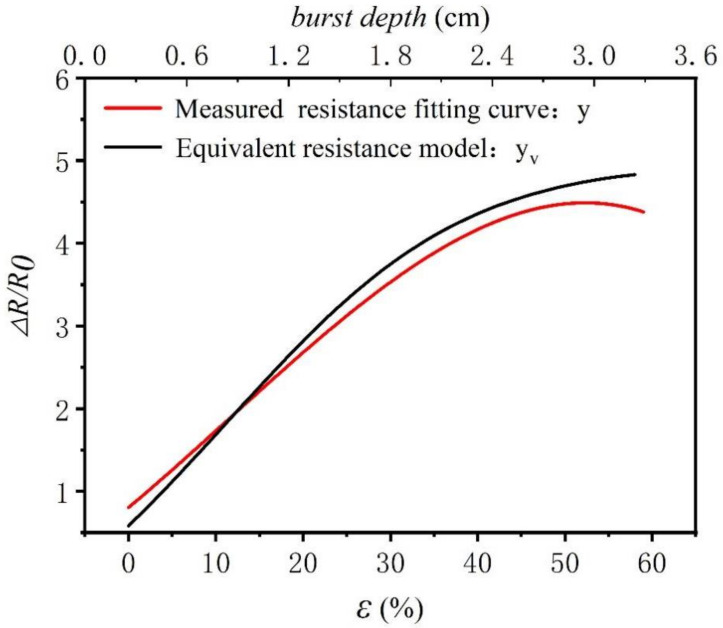
The theoretical model of equivalent resistance and the results of the burst test when the three-dimensional surface was strained.

From the analysis in Figure 11, there is a power function relationship between equivalent resistance and burst depth. As the burst depth increases, the resistance change rate tends to be flat after rapid increases in resistance. This change rule better explains three-dimensional curved surface strain-resistance changes, which are consistent with the results of the curved surface resistance test. Therefore, a theoretical formula of equivalent resistance can explain the three-dimensional curved surface strain resistance change rule.

## 4. Conclusions

In this study, two in-plane equivalent resistance models of knitted strain sensors and a three-dimensional equivalent resistance were presented. It is verified that these three resistance models can predict the resistance change of knitted strain sensors. Based on the in-plane strain, a macro–micro equivalent resistance model and topological equivalent resistance model are established. The macro–micro equivalent resistance model is a combination of two structural models, based on length resistance from a macro point of view and loop transfer from a micro point of view. The topological equivalent resistance model is based on a single loop and considers the superimposed resistance between the loops.

Comparing the in-plane tensile experimental data with the resistance models, the topological resistance model has a higher prediction and fitting accuracy, but the calculation is more complex, which is suitable for small-area knitted sensor applications. The macro–micro equivalent resistance model is simple and quick to calculate, suitable for large-area knitting sensor applications. Based on volume resistivity, a three-dimensional curved surface equivalent resistance model is established, which has a significant positive correlation effect on the change of the curved surface strain resistance. Furthermore, the results strongly suggest that the resistance change occurrence is directly related to the length resistance, loop transfer, and burst depth of the knitted sensor.

## Figures and Tables

**Table 1:** ANOVA for the in-plane tensile cycle stretching experiment.

	Sum of Squares	df	Mean Square	*F*	*p* Value
Regression	6081.70	50	121.63	0.688	0.95
Residual	4,326,928.20	24471	176.81		
Total	433,009.90	24521			

**Table 2:** Parameter estimates for in-plane tensile equivalent resistance model.

	Unstandardized Coefficients		Standardized Coefficients	*t*	*p*	$R^{2}\;$	*F*
	*B*	Std. Error	*Beta*				
Constant	−0.235	0.026	-	−8.959	0	0.975	39,060.82
Fitting resistance	1.084	0.005	0.987	197.638	0		

**Table 3:** Parameter Estimates for topology equivalent resistance model.

	Unstandardized Coefficients		Standardized Coefficients	*t*	*p*	*R^2^*	*F*
	*B*	Std. Error	*Beta*				
Constant	−15.801	0.082	-	−92.94	0	0.99	43,544.393
Fitting resistance	16.965	0.081	0.99	208.67	0		

**Table 4:** Linear regression analysis of the three-dimensional equivalent resistance model and the actual resistance.

	Unstandardized Coefficients		Standardized Coefficients	*t*	*p*	*R^2^*	*F*
	*B*	Std. Error	*Beta*				
Constant	0.218	0.005	-	41.265	0	0.867	366,274.18
Fitting resistance	0.888	0.001	0.99	605.26	0		

## Data Availability

Not applicable.
